# The complex rostral morphology and the endoskeleton ossification process of two adult samples of *Xiphias gladius* (Xiphiidae)

**DOI:** 10.1111/jfb.15069

**Published:** 2022-05-16

**Authors:** Ugo E. Pazzaglia, Marcella Reguzzoni, Marco Saroglia, Renata Manconi, Guido Zarattini, Mario Raspanti

**Affiliations:** ^1^ Department of Specialità Chirurgiche, Scienze radiologiche e Sanità Pubblica University of Brescia Brescia Italy; ^2^ Department of Medicina e Chirurgia University of Insubria Varese Italy; ^3^ Department of Biotecnologie e Scienze della Vita University of Insubria Varese Italy; ^4^ Department of Veterinary Medicine, Zoology Lab University of Sassari Sassari Italy

**Keywords:** anosteocytic bone, bone remodeling, osteichthyes, osteogenesis, rostrum morphology, swordfish

## Abstract

The authors studied the morphology of the upper and lower jaws, vertebrae and dorsal‐fin rays of the teleost fish *Xiphias gladius* to analyse the skeletal architecture and ossification pattern. The analogies and differences among these segments were investigated to identify a common morphogenetic denominator of the bone tissue osteogenesis and modeling. The large fat glands in the proximal upper jaw and their relationship to the underlying cartilage (absent in the lower jaw) suggested that there is a mechanism that explains rostral overgrowth in the Xiphiidae and Istiophoriidae families*.* Thus far, the compact structure of the distal rostrum has been interpreted as being the result of remodeling. Nonetheless, no evidence of cutting cones, scalloped outer border of osteons and sequence of bright–dark bands in polarized light was observed in this study, suggesting a primary osteon texture formed by compacting of collagen matrix and mineral deposition in the fat stroma lacunae of the bone, but without being oriented in layers of the collagen fibrils. A similar histology also characterizes the circular structures present in the other examined segments of the skeleton. The early phases of fibrillogenesis carried out by fibroblast‐like cells occurred farther from the already‐calcified bone surface inside the fat stroma lacunae. The fibrillar matrix was compacted and underwent mineral deposition near the previously calcified bone surface. This pattern of collagen matrix synthesis and calcification was different from that of mammalian osteoblasts, especially concerning the ability to build a lacuno‐canalicular system among cells. Necrosis or apoptosis of the latter and refilling of the empty lacunae by mineral deposits might explain the anosteocytic bone formation.

## 1 INTRODUCTION

Among the very large number of living fish species only those of the two families Istiophoridae and Xiphiidae are known to develop an extraordinarily long upper jaw (the rostrum), which can be 20 times or more longer than the lower jaw. This particular feature has attracted the attention to rostral morphology, biomechanics and function in hunting and feeding (Atkins *et al*., 2014; Carey, 1982; De Metrio *et al*., 1997; Domenici *et al*., 2014; Fierstine, 1997; Fierstine & Voigt, 1996; Habegger *et al*., 2015, 2019; Mc Gowan, 1988; Schmidt *et al*., 2019; Videler *et al*., 2016). In particular, the rostrum of *Xiphias gladius* (Xiphiidae) and other species of the Istiophoridae family has been extensively studied and presented as clear examples of anosteocytic bone osteonal organization in teleost fishes (Atkins *et al*., 2014; Currey & Shahar, 2013; Habegger *et al*., 2015; Poplin *et al*., 1976; Shahar & Dean, 2013).

Since the first observations by Koelliker (1859), the presence of bone devoid of osteocytes in living teleosts has been reported using the terms “acellular” or “anosteocytic” bone (Moss, 1961a, 1961b, 1963; Weiss & Watabe, 1979). Nonetheless, a more recent study in the zebra fish *Danio rerio* has shown that both anosteocytic and cellular bone can be observed in the skeleton of this fish and also within the same skeletal element, suggesting that a transition from the cellular to the acellular bone type occurred (Weigele & Franz‐Odendaal, 2016). The process underlying anosteocytic bone osteogenesis was considered to be basically similar to that of cellular bone derived from a chondroid‐type tissue (chondroid osteogenesis) or from the periosteum (periosteal osteogenesis), and the development of acellularity was considered to have developed from the withdrawal of osteoblasts that do not become entrapped as osteocytes (Ekanayake & Hall, 1987; Moss, 1961a; Weiss & Watabe, 1979) or from cells dying once entrapped because conditions for survival are not given and the lacunae are refilled by the calcified matrix (Moss, 1961b; Ofer *et al*., 2019). In addition to the mechanism underlying anosteocytic bone formation, a number of questions concerning structure, calcification and vascular supply of the anosteocytic bone; the distribution of the two types in teleost species; and the developmental phases in the individual fish still need to be answered. Phylogeny in teleost taxa has been used to tackle the issue of cellularity distribution (Lynne & Parenti, 1986), as also extensively reported in a recent review by Davesne *et al*. (2019). In that paper, the authors state that “the broad consensus on the two statements (i) that cellular bone is the plesiomorphic condition for teleosts, actinopterygians and osteoichthyans in general and (ii) that acellular bone is found in ‘advanced’ or ‘higher’ teleost groups are imprecise and potentially misleading.” These authors also suggested taking a phylogenetic approach to distinguish the role of adaptation from that of the phylogenetic history in the distribution of bone types among species through large‐scale molecular analyses that are now available (Betancur *et al*., 2017; Hughes *et al*., 1994; Near *et al*., 2012).

Furthermore, fundamental knowledge of bone resorption and remodelling is also needed to understand teleost bone development (Witten *et al*., 2000, 2001). Witten and Huysseune (2009) emphasized this point in their review, in contrast to the previous view, that teleost bone is a metabolically inactive tissue, not subjected to remodelling, a view based on old publications reporting the absence of multinucleated osteoclasts and failure to respond to parathyroid hormone stimulation (Ingleton *et al*., 1995; Simmons, 1971). These authors further stated the importance of accurately considering both the differences in and similarities to mammalian morphology and metabolism. This task is certainly arduous due to the large number of species in both fish and mammalians but also to the specific differences present in species of the latter class of vertebrates. In taxa with cellular bone, the osteocytes are the dominant cellular components representing up to 95% of all the cells (Hall, 2005); they are derived from the osteoblasts that have remained embedded in the matrix produced by the same cells (Franz‐Odendaal *et al*., 2006) and can survive in the calcified matrix environment because the osteoblast–osteocyte transformation and the calcification of the extracellular matrix have progressed simultaneously with the formation of the lacuno‐canalicular network (Pazzaglia *et al*., 2010, Pazzaglia & Congiu, 2013).

In this paper, the skeletal histo‐morphology of *X. gladius* Linnaeus, 1758, was examined, with focus on the basic processes of osteogenesis and remodeling. The arbitrary choice to study this fish as an experimental model was based on the following considerations: (a) *X. gladius* is the only species of the Xiphiidae family and is undisputedly a bony fish; (b) its high degree of skeletal specialization such as rostral development; (c) the availability of sufficiently large‐sized, full‐grown subjects capable of providing proper bone samples for processing and precise orientation of histological slides and specimens for scanning electron microscopy (SEM) observation; and (d) recent reports suggesting rostral remodeling (Atkins *et al*., 2014; Habegger *et al*., 2015, 2019; Shahar & Dean, 2013).

The following skeletal elements were examined: the upper and the lower jaws, the vertebrae and the dorsal‐fin rays. The aims of this study were (a) to highlight the histology and the mechanism underlying growth in rostral length, (b) to compare the structural layout of the upper and lower jaws, (c) to document anosteocytic bone formation mechanism in the examined skeletal segments and (d) to search for evidence of osteonal secondary remodelling in the distal rostrum.

## 2 MATERIALS AND METHODS

### 2.1 Samples

For this study, bones were obtained from two swordfishes (2.90 and 3.60 m in length, respectively) captured in the Sardinian Sea (Western Mediterranean). The study complied with all ethical requirements of the *Journal of Fish Biology* and local authorities. Specimens were purchased from commercial sources (Mercato Ittico Milano, Milan, Italy); animal welfare laws, guidelines and policies were not applicable. The fishes were received within 24 h of capture, and small specimens of the tissues to be examined were dissected and immediately fixed in 10% buffered formaldehyde solution for 3 weeks and then conserved until processing in a 4% solution of the same fixative. This procedure provided a satisfactory fixation for both cells and extracellular matrix histology and SEM.

### 2.2 Preparation and selection of anatomical specimens

The rostrum was cut transversally with a band saw into five segments of *c*. 40 cm in length, with short 5 cm long segments between the longer samples. The proximal segment included the proximal rostral cone and part of the frontal bones. X‐rays of the long segments of rostrum were taken in antero‐posterior (a‐p) and lateral projections; the short segments were further sectioned in slices of 5 mm thickness to obtain X‐ray images in the transverse projection. Computed tomography (CT) was performed separately on the five long rostral segments from the basal cone to the tip using a NewTom Cone Beam CT equipment (New Tom, Verona, Italy). The two branches of the lower jaw were first divided and separated from the tip; then X‐rays were taken in the a‐p and lateral projections. The first five proximal vertebrae were dissected, radiographed and examined with CT. The dorsal‐fin rays were radiographed and then sectioned at the base.

### 2.3 Macromorphology and histo‐morphology

The bone specimens of the upper and lower jaws, vertebrae and fin rays were reduced to smaller specimens and sectioned for histology using a low‐speed, diamond circular saw (Buheler Ltd., Lake Bluff, Illinois, USA). Subsequently, 2 mm thick sections were processed for SEM observation; 500 μm thick sections were further reduced to *c*. 300 μm by manual grinding; the sections were then polished, ultrasonicated and stained and undecalcified with toluidine blue or using the Von Kossa method before being examined in reflected and transmitted light Using a low‐power stereomicroscope (Olympus SZX 7, Japan). Other specimens were decalcified in a solution of acetic and hydrochloric acid (2% CH_3_HCOOH/2% HCl) for 30 days, dehydrated in increasingly concentrated ethanol–water solutions and embedded in paraffin. Transverse and longitudinal 7 μm thick sections were cut with a sledge microtome and stained with haematoxylin–eosin, May‐Grunwald‐Giemsa, periodic acid‐Schiff, Osmium and Alcian blue.

### 2.4 Morphometry

Examination using circularly polarized microscopy was carried out to evaluate the number and density of the following osteon types: (a) full dark, (b) full bright and (c) alternate sequence of bright and extinct bands (Bromage *et al*., 2003). Transverse, decalcified thin sections (stained with haematoxylin–eosin) were cut at three levels of the rostrum at a distance of −10, −20 and −30 cm from the tip. Fifty randomly selected rectangular fields (434.52 × 325.04 μm) were acquired for each level at 100× magnification using a Colour View IIIb digital camera (Soft Imaging System GmbH, Munster, Germany) in circularly polarized light microscopy. Only osteons not intersected by the field borderline were counted and assigned to the osteon typology as reported earlier. Statistical analysis could not be applied to the distribution of osteon typology because all resulted in the “all dark” class. The number and density of osteons among the levels were compared with MedCalc programme (MedCalc Software, Ostend, Belgium). Differences between groups were assessed using *t*‐test; a probability of *P* < 0.05 was considered statistically significant.

### 2.5 Scanning electron microscopy

Two different preparation techniques were applied to examine the laminar structures on the outer surface of the lower jaw (a) and the compact tissue of the distal rostrum (b): (a) Bone specimens were kept for 30 days in a 40% oxygen peroxide solution to remove the epidermis and the underlying soft tissues and then were repeatedly washed and sonicated in a bath of buffered saline solution (pH 7.4). They were then subjected to a slight, superficial decalcification in a 6% Na_3_PO_4_ solution (pH 9.1) for 1 min at 15°C to display the collagen scaffold, and then they were routinely processed for SEM observation. (b) The transverse and longitudinal sections of the distal rostrum (2 mm thick) were manually ground with the finest sandpaper (p 5000) and then polished on a smooth blackboard surface. They were ultrasonicated in a bath of buffered saline solution, pH 7.4, until completely clear of mechanical manipulation debris, and then kept in a 6% Na_3_PO_4_ solution (pH 9.1) at room temperature for 1 min. The specimens were dehydrated in increasingly concentrated ethanol solutions, critical point dried in a CO_2_ environment and coated with a thin layer of gold in a sputter coater (Quorum Techn, Laughton, UK). For viewing, the authors used a Philips XL30 scanning electron microscope (Philips, Eindhoven, the Netherlands).

## 3 RESULTS

### 3.1 Macro‐ and histo‐morphology

#### 3.1.1 Upper jaw (rostrum)

**FIGURE 1:**
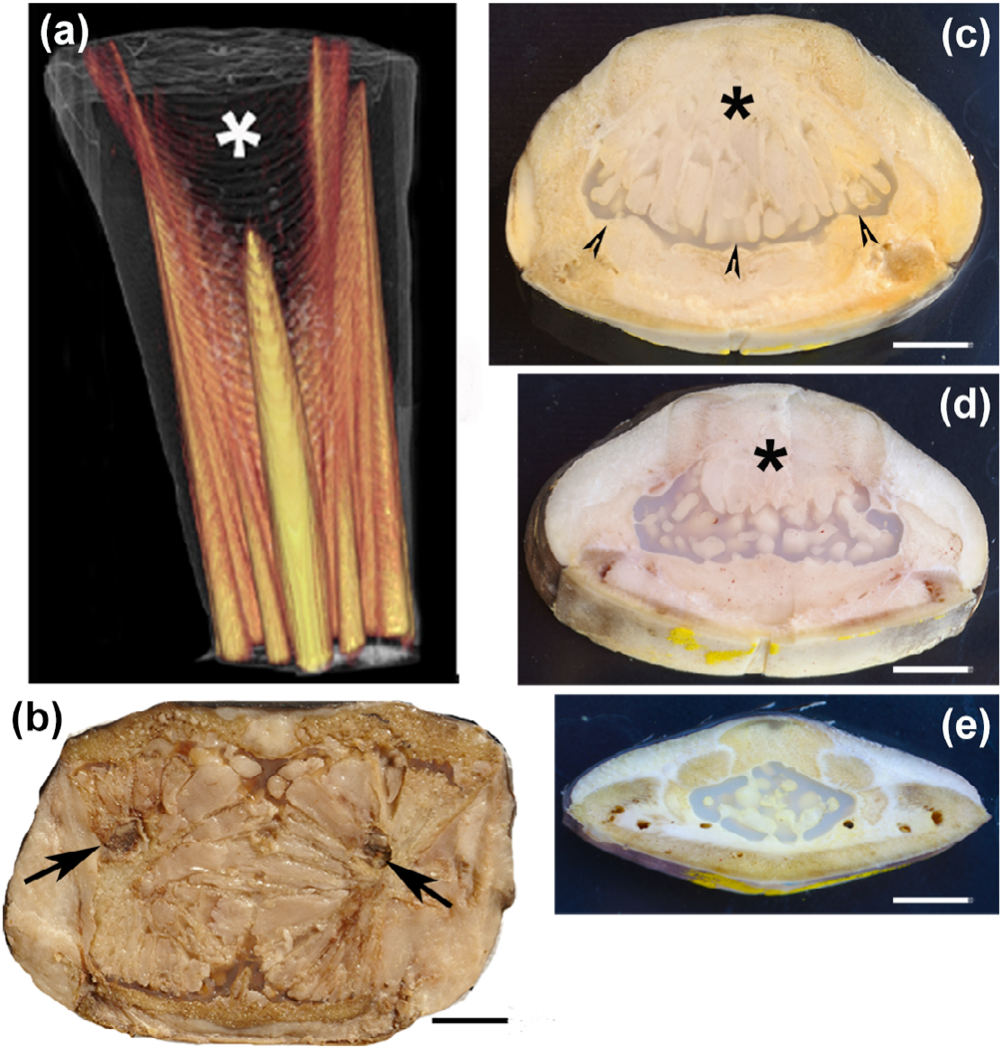
*Xiphias gladius*. Proximal cone of the rostrum CT 3D reconstruction and transverse sections of the upper jaw. (a) CT (computed tomography) 3D reconstruction showing the ossification pattern of the dorsal rostrum forming distinct columns of calcified matrix which extend distally. The uncalcified zone in the centre (asterisk) corresponds to the fat gland; (b–e) fresh tissue, unstained transverse sections (10 mm thick) photographed in reflected light at different levels of the cone and proximal rostrum (bar = 10 mm): (b) symmetrical fat glands with the lobular pattern developing around a central, vasculo‐neural axis (arrows). Below, the flat bony floor of the upper jaw is evident; (c) more distal transverse section showing a thin layer of cartilage‐like material (arrowheads) below the fat gland; (d) fat bubbles are pushed inside the cartilage‐like matrix from the above gland (asterisk); (e) more distal section showing flattening and size reduction in the rostrum with formation of distinct columns dorsally. The cartilage‐like structure (reported as “cartilage” by Habegger *et al*., 2015) fills the central rostral space; below is evident the flat bony floor of the upper jaw

**FIGURE 2:**
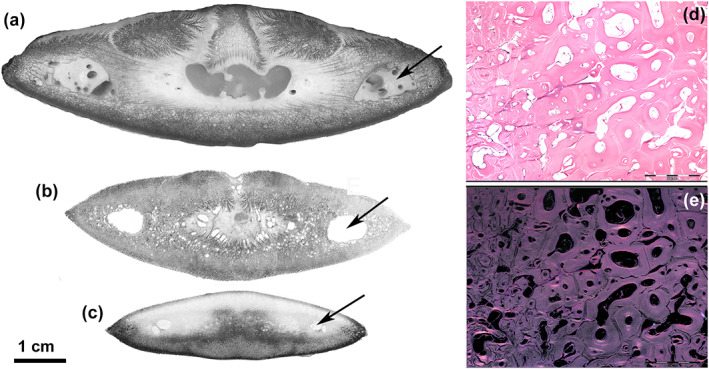
*Xiphias gladius*. Transverse sections of the distal rostrum at different levels. (500 μm thick sections, toluidine blue reflected light, bar = 10 mm). (a) Still evident is the central cartilage‐like body with a few fat bubbles inside. Two symmetrical large tunnels on both sides (arrow) contain a dense fat stroma, which also surrounds the central cartilage and fills the spaces between the latter and the dorsal columns. Ossification advances in the fat stroma with finely arborized laminae. The basal layer is formed by compact bone matrix; (b) central cartilage‐like body is reduced to a thin cylinder in the centre surrounded by fat tissue. The dorsal columns formed a single, dorsal layer with a median groove, whereas ossification advances centripetally with small arborizations. Small lacunae can be observed in the calcified matrix; (c) peripheral rostrum shows a compact peripheral layer of calcified matrix; the size of the lateral tunnels is reduced, small lacunae in the central area still evident; (d, e) distal rostrum (10 μm thin, decalcified sections, haematoxylin–eosin in bright field and circularly polarized light, 100×, bar = 200 μm). Transverse sections showing a mixed texture of densely packed laminar and circular structures around oval or round lacunae. Bone is anosteocytic in both, and in circularly polarized light (e) all display the “dark” type

The floor of the rostrum consisted of a layer of densely mineralized tissue, whereas the roof was composed of distinct calcified columns that delimited a central, vacuolated mass of soft tissue (Figure 1a). The rostrum of *X. gladius* in the distal progression had a blade‐like shape maintaining a different pattern of a compact ventral layer and distinct dorsal columns up to about half of its length and then merged and fused with the lower layer to form a closed flattened ring (Figure 2c–e).

The macro‐imaging and histology of a series of rostral cone transverse sections (10 mm thick) showed two large and symmetrical fat glands that almost completely filled the sectional area between the bony floor and roof of the upper jaw bone. The glandular lobules displayed a radial layout around two symmetrical canals *c*. 4 mm in diameter (Figure 1b); the next section showed a single glandular body, with vertical lobules abutting downward on a thin layer of cartilage‐like tissue (Figure 1c,d); and in the following section the area of the gland became progressively smaller while the underlying mass of cartilage‐like tissue filled the rostral centre showing fat bubbles inside (Figure 1d,e). More distally, the cartilage‐like tissue disappeared about halfway down the length of the rostrum (Figure 2a–c). The cartilage‐like tissue had a gel‐like consistency softer than the fat bubbles, and this area as well as the embedded bubbles decreased advancing forward in the rostrum. About halfway down the length of the rostrum, the whole section consisted of calcified bone matrix (Figure 2c). On histology, this cartilage tissue showed a homogeneous matrix weakly stained by haematoxylin and Alcian blue, with small flat or elongated cells inside the lacunae. A similar tissue, but without fat bubbles, could be observed in the Meckel cartilage of the lower jaw in the same fish, suggesting that this was embryonic cartilage. The bubbles embedded in the matrix were stained by Osmium, not by Alcian blue; the matrix was evenly stained by Alcian blue and faintly by Sirius red, which was concentrated on the bubbles' borders (Supporting Information Figure FIGURE  S9). The rostral architecture changed from the basal cone to the tip, with the central cartilage disappearing and the dorsal ossification columns and the basal bony layer fusing together (Figure 2c). Two large tunnels ran on each side and along the whole length of the rostrum; they contained a fat stromal tissue with large arteries and myelinic nerves inside (Figure 2a; Supporting Information Figure S8a,b). The same fat stroma filled all the interstitial bone spaces in the rostrum.

**FIGURE 3:**
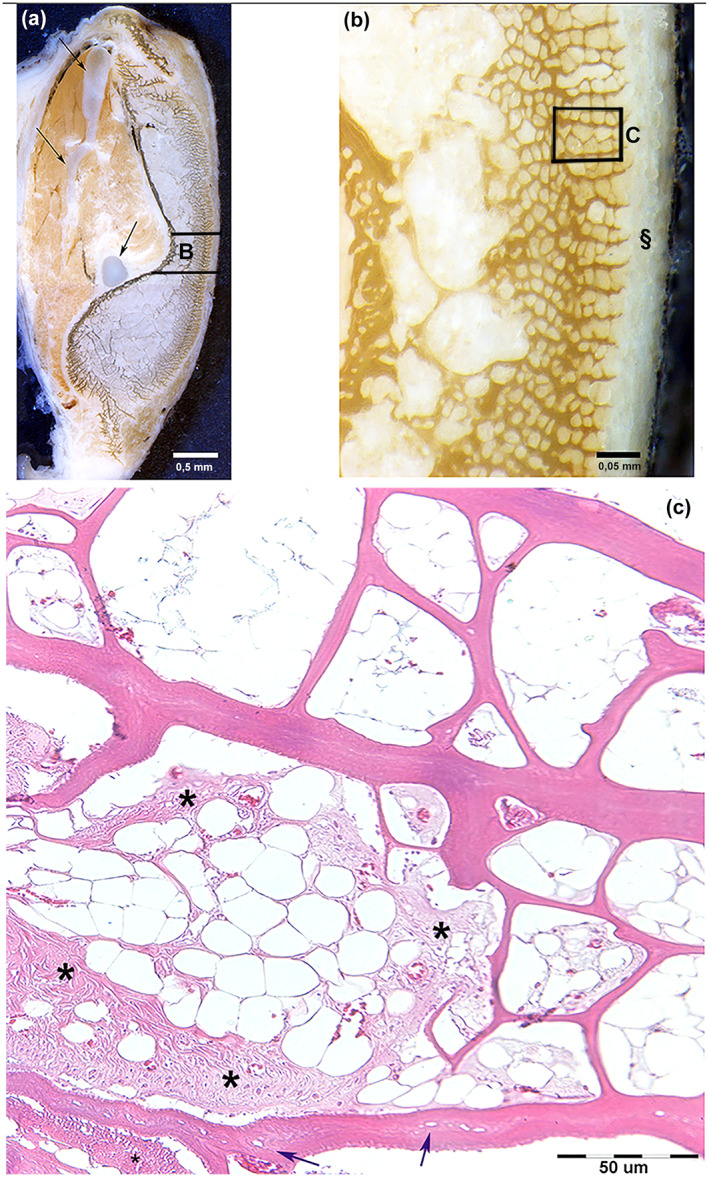
*Xiphias gladius*. Lower‐jaw left branch. (a) Toluidine blue in reflected light, transverse 500 μm thick section, bar = 0.5 mm. Bean‐like shape of the bone showing the inner and outer bone wall: the inner with a compact texture, the outer consisting of parallel, calcified laminae connected by thin transverse septa. The Meckel cartilage (arrows) and muscles are evident on the inner surface; the bone grows at the upper and lower poles with the formation of new laminae with the same appearance of the latter; (b) details of the central outer part (sector B of A, 500 μm thick section, toluidine blue in reflected light, bar = 0.05 mm). The outer surface is covered by the sub‐epidermal fat tissue (§). The inner wall presents a more compact texture of the calcified matrix. The external laminae and the thin septa delimit lacunar spaces filled by a cellular‐adipose stroma. (c) Decalcified section, 10 μm thick, haematoxylin–eosin, bar = 50 μm. Random distribution of “resting” and “active” zones in the cellular‐adipose stroma of lacunae; the latter show hypertrophic capillaries and fibrillogenesis (asterisks). The bone of laminae and septa is anosteocytic; the lamina far down still shows empty cellular lacunae (arrows)

Ossification of the dorsal columns of the proximal rostrum, as shown by thick section histology, progressed into the fat stroma with a branching pattern (Figure 2a,b). The compact bone texture of the proximal basal layer was proximally transformed into a close compact bone ring distally (Figures 1e and 2b,c). The thin‐section histology of the proximal column ossification showed laminae of the anosteocytic bone protruding into the fat stroma without any evidence of a periosteal membrane, exactly similar to that described in the lower jaw (Figure 3a,c). Both the proximal rostral floor and the distal cortical ring showed a compact texture of densely packed circular and laminar structures that had developed around the small vessels and capillaries of the fat stroma. The layout of this calcified tissue suggested an osteonal architecture (Figure 2d,e), however, with some peculiarities: (a) absence of osteocytes, (b) absence of the peripheral cement line, (c) absence of cutting cones and (d) absence of the bright–dark band sequence on circularly polarized light microscopy (Figure 2e).

All the osteons examined at levels −10, −20 and −30 cm from the rostral tip were of the “all dark” type. The differences between the mean osteon number and field among these fields were not significant, whereas the mean osteon density was significant (*P* < 0.001) between levels −30/−20 and −20/−10 (Table 1).

**TABLE 1:** All the osteons at the three examined levels of the distal rostrum (−30, −20 and −10 mm from the tip) correspond to the “dark” type in circularly polarized light microscopy. mean number and density (s.d) among levels is significantly higher  < 0.001) between levels −30/−20 and

Osteon type	Osteon mean density n mm^–2^ ±s.d.		
Level from tip (cm)	−30 cm	−20 cm	−10 cm
Examined field number	50	50	50
Concentrical bright–dark bands	0	0	0
All bright	0	0	0
All dark	61±6	128±6.5	176±6
	*P* ≤ 0.001		
		*P* ≤ 0.001	

*Notes*: All osteons at the three examined levels of the distal rostrum (−30, –20 and –10 cm) correspond to the “all dark” type.

#### 3.1.2 Lower jaw

**FIGURE 4:**
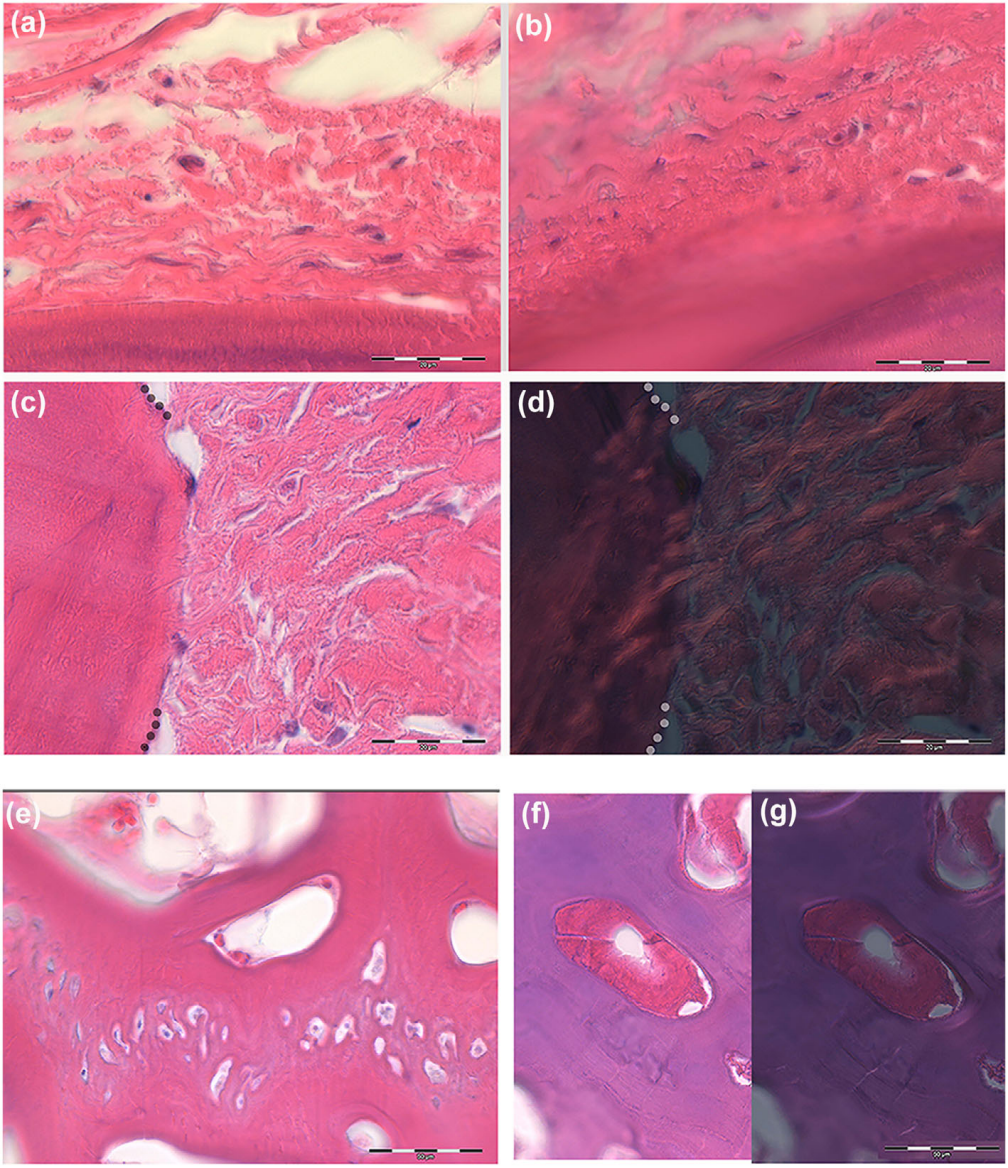
*Xiphias gladius*. Lower‐jaw outer wall (haematoxylin–eosin in bright and polarized light, bar = 20 μm): (a, b) (haematoxylin–eosin in bright field, bar = 20 μm) progression of collagen fibres compacting on the anosteocytic bone surface with fibroblast‐like cells embedded in the matrix; (c, d) (haematoxylin–eosin in bright and polarized light, bar = 50 μm). Borderline (dotted lines) between the earlier‐calcified anosteocytic bone and the outer, not‐yet‐calcified, compacted matrix; birefringence documents the fibre bundle continuity between the outer and the inner calcified matrix (Sharpey‐like fibres); (e) (haematoxylin–eosin in bright field, bar = 50 μm) histology of a lamina of anosteocytic matrix with cellular zone of fibroblast‐like cells embedded in the calcified matrix. There is no evidence of cutting cones or scalloped resorption pits around these cells; (f, g) (haematoxylin–eosin in bright field and polarized light, bar = 50 μm) circular matrix deposits inside lacunar spaces of the bone. They do not show the distinctive traits of secondary osteons (cutting cone scalloped profile and peripheral cement line). In polarized light there is no evidence of bright–dark band sequence

The lower jaw was formed by two elongated branches converging and merging in an anterior pointed tip (Supporting Information Figure S7c). The transverse, undecalcified thick sections were stained by Von Kossa; the corresponding decalcified thin sections showed a bean‐like shape with a concave inner and a convex outer surface: the inner surface was covered by jaw muscles, with the Meckel cartilage (remnant of the embryonic first arch cartilage) that ran like a cord along the whole length of the jaw; the outer surface was covered by sub‐epidermal fat without interposition of a membrane (Figure 3a). At higher magnification, the transverse section showed the compact texture of the inner cortex and a pattern of the outer cortex formed by a sequence of parallel, pointed laminae sticking out in the sub‐epidermal fat (Figure 3b); these laminae were connected by thin transverse septa. Lacunae delimited by laminae and septa were filled by fat tissue and vascularized by capillaries. Scattered aggregates of fibrous connective tissue formed inside the fat stroma of the lacunae (Figure 3c). The upper and lower poles of each branch showed less‐ordered laminae that developed outwardly in the fat stroma (Figure 3a). Most of the laminae and septa of the lower‐jaw outer surface consisted of compact anosteocytic bone matrix, with some showing an irregular distribution of empty or cellular lacunae usually positioned in the central band of the lamina (Figures 3c and 4e). Fibrillogenesis in the lacunae ensued near the already‐calcified bone surface without an ordered layout of the fibroblast‐like cells, which is remarkably different from the regular rows of osteoblasts in the active phase of osteoid apposition. The fibrillar matrix also became compacted, embedding the fibroblast‐like cells in the layer adjacent to the previously mineralized surface (Figure 4a,b). The mineral deposition on the compacted fibril layer enlarged the thickness of the lamina as documented by the continuity of collagen fibril bundles between the previously calcified and the still‐uncalcified, recently compacted band of tissue (Figure 4c,d). A few scattered lacunae with an embedded cell or empty could be observed in the general context of the anosteocytic matrix of the lamina (Figure 4e).

The inner side of the lower jaw was thinner and had a more compact texture than the mineralized matrix of the outer side, with circular or oval lacunae being distributed there. Some of the latter contained deposits of a not‐yet‐calcified matrix, suggesting a circular osteon‐like pattern (Figure 4f), however, with no evidence of a fibrillar material orientation in polarized light (Figure 4g).

#### 3.1.3 Vertebrae

**FIGURE 5:**
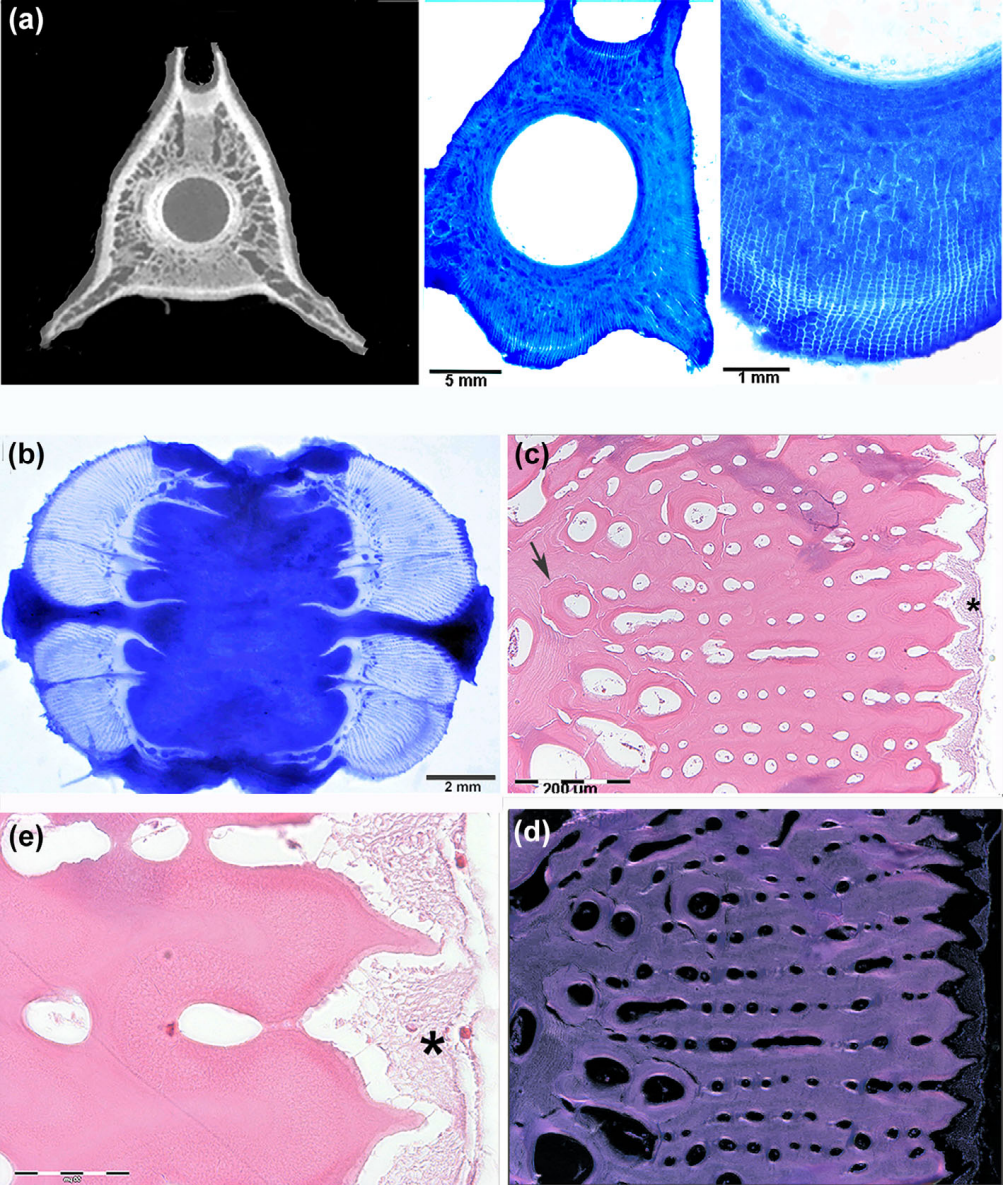
*Xiphias gladius*. Spine third vertebra and dorsal‐fin fourth ray. (a) [CT (computed tomography) coronal section marginally intercepting the second to third intervertebral disk and 500 μm thick, undecalcified coronal sections, toluidine blue in reflected light, bar 5 and 1 mm]. The vertebral body shows a central, spongiform texture with a more compact layer around the intervertebral disk. The coronal section evidences the right and left ventral apophyses and the neural groove dorsally. The corresponding histological sections show a lacunar pattern of the central calcified matrix and a peripheral, laminar layout not different from that of the upper and lower jaws; (b) (coronal thick sections of the dorsal‐fin fourth ray, 500 μm thick, undecalcified section, toluidine blue in reflected light, bar = 2 mm). The ray is formed by four ossification centres with densely packed, parallel laminae springing from a compact base and oriented outward. Shortest, thick and pointed apophyses emerge from the base towards the mid‐longitudinal line of the ray; (c, d) (transverse decalcified thin sections of the ray sub‐unit, haematoxylin–eosin in bright field and polarized light, bar = 200 μm) densely packed laminae of anosteocytic bone separated by round, small lacunae with a parallel alignment. Some lacunae in the basal layer develop the circular pattern already observed in rostrum typical of primary osteons (arrow). The corresponding polarized light field shows the “dark” pattern in both laminae and circular structures; (e) (transverse decalcified thin section of the ray sub‐unit, haematoxylin–eosin in bright field, bar = 50 μm) fibrillogenesis with the same characters of the other endoskeleton segments can also be observed in the sub‐epidermal fat tissue between the tips of the laminae (asterisk)

Spherical intervertebral disks were interposed between adjacent vertebrae, with a transparent tissue enclosed by the notochord sheath. In the coronal section, the vertebral body had a three‐cornered shape with two symmetrical basal processes and the neural groove at the top. The peripheral border showed a layer of denser, calcified matrix, whereas the inner body mass had a lacunar texture (Figure 5a). The corresponding 300 μm thick undecalcified sections showed the same ossification pattern, with the sequence of parallel laminae bound by transverse septa all around the outer bone border and a texture of laminar and circular structures in the centre (Figure 5a).

#### 3.1.4 Dorsal‐fin rays

The first and second rays of the dorsal fin were short and underdeveloped; the third was the longest one and with a single calcified axis. The other fin rays from the fourth onwards had a single basement which fanned out in four calcified filaments covered only by tegumental tissue (Supporting Information Figure S10a). The transverse section of the fin‐ray basal segment showed four distinct ossified nuclei separated by fat cellular stroma. Each of these was formed by a system of densely packed laminae springing from a compact base and oriented outwardly. Shortest and thick/pointed apophyses emerged from a compact base towards the mid‐longitudinal line of the fin ray (Figure 5b).

Histology showed a texture of parallel, densely packed and pointed laminae. A row of aligned small, circular or occasionally long and narrow lacunae suggested a structural layout similar to that observed in the dorsal ossification columns of the rostrum, in the low jaw and in the vertebrae. The small lacunae contained fat stroma and capillaries. The basal layer had a compact texture with larger, roundish lacunae with osteon‐like circular structures (Figure 5c). Bone had an anosteocytic texture without evidence of bright–extinct bands in polarized light (Figure 5d). Fibrillogenesis was observed only in the external fat stroma between the tips of the densely packed laminae (Figure 5e), suggesting that the lengthwise growth of the fin ray could develop at this level, whereas a similar mechanism was not detected at the base of the fin ray.

### 3.2 Scanning electron microscopy

**FIGURE 6:**
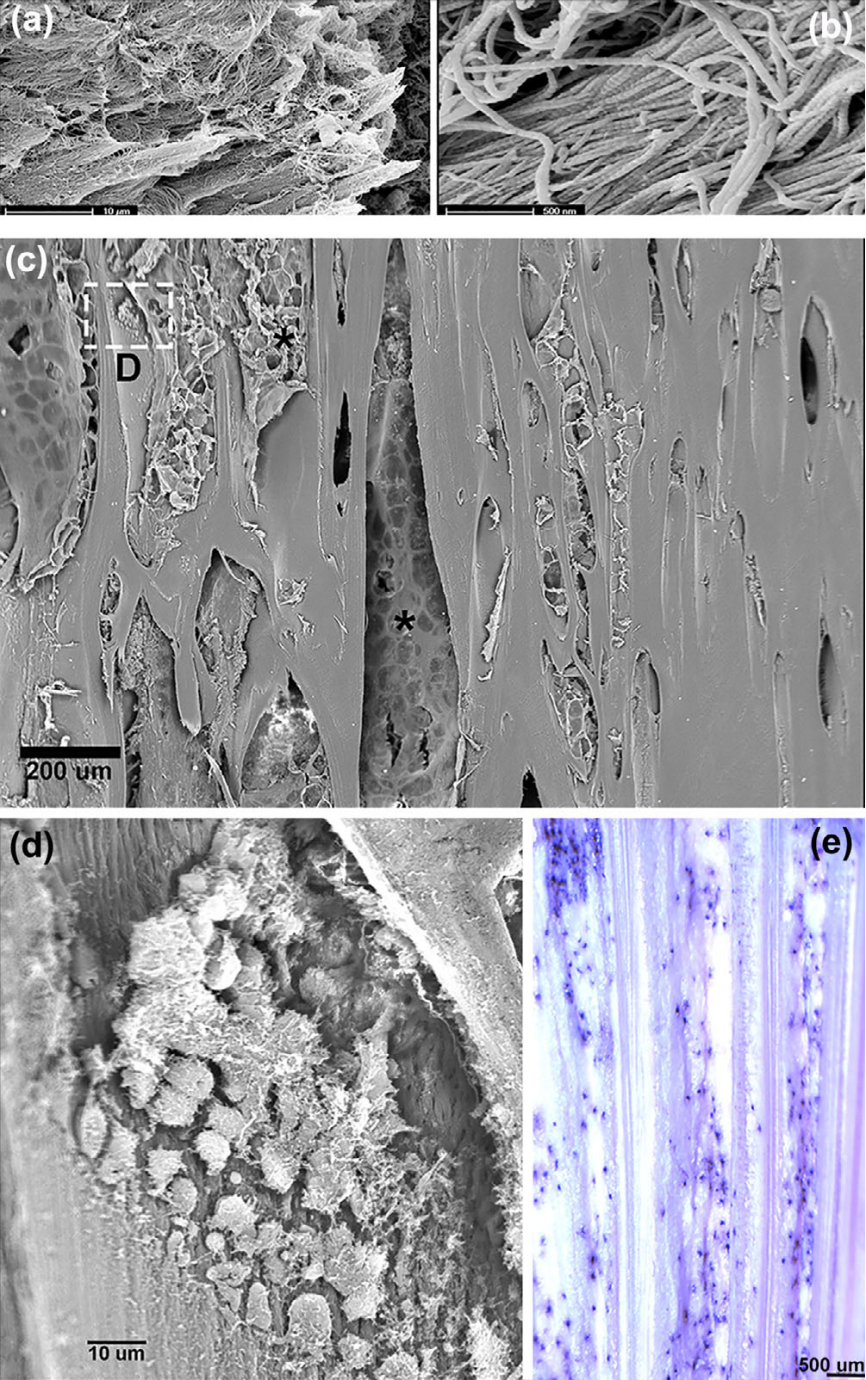
*Xiphias gladius*. Lower‐jaw outer wall and rostrum [SEM (scanning electron microscopy)]. (a) Overlapping laminae of the outer wall are displayed by removal of tegument, sub‐tegumental fat and weik etching with NaOH solution; they show a densely packed layout of collagen fibres ending with sharpened tips (bar = 10 μm); (b) higher magnification shows the collagen fibril period (bar = 500 μm); (c) distal rostrum longitudinal section displays the circular structures shown by light microscopy transverse sections and the fat stroma of the lacunae (asterisk) (bar = 500 μm); (d) higher magnification of the outlined area in c showing a sheet of smaller cells than adipocytes in the process of deposing matrix on the inner surface of the lacuna (bar = 10 μm); (e) distal rostrum undecalcified, thick longitudinal section light, toluidine blue in reflected light (bar = 500 μm) showing the longitudinal layout of the circular structures. The toluidine‐stained dots correspond to the cells of the SEM image D

SEM observation confirmed the two calcified matrix aggregation patterns demonstrated by light microscopy study of the *X. gladius* skeleton. (a) The laminar pattern was characterized by flat and pointed plates aggregated in a stratified structure (Figure 6a). The latter was formed by a compact network of collagen fibrils as substantiated by the documented evidence of the period at higher magnification (Figure 6a). (b) The circular pattern had densely packed structures (osteon‐like) that represented the widespread structural layout of the distal rostrum, where collagen matrix had been deposed on the inner surface of the lacunae filled by the fat stroma (Figure 6c). Collagen was synthesized by small, globular or flattened cells of *c*. 10 μm in diameter, easily distinguishable from the adipocytes of the stroma for both their size and shape (Figure 5d). They could be also demonstrated in undecalcified, 100 μm thick sections stained with toluidine blue and observed by light microscopy (Figure 5e). No evidence of collagen fibril plywood layout, resorption pits or cutting cones was found in transverse and longitudinal sections examined with SEM in either the rostrum or lower jaw.

## 4 DISCUSSION

The higher degree of skeletal specialization of *X. gladius* is represented, in particular, by the considerable development of length of the upper jaw, whose hard and stiff rostral structure is known to consist of anosteocytic bone. Nonetheless, several aspects concerning its morphology, growth and function are still uncertain and being debated. The most recent studies of the rostrum of the Xiphidae and Istiophoridae families focused on the biomechanics and the function of this structure but did not include an in‐depth study of basic histo‐morphology and SEM ultrastructure (Atkins *et al*., 2014; Fierstine & Voigt, 1996; Habegger *et al*., 2015, 2019; Schmidt *et al*., 2019). The complex anatomy of the swordfish rostrum provides a model for comparing the morphology with other bones of this fish and for investigating the particularities of its structure and development. De Metrio *et al*. (1997) examined the anterior skull of *X. gladius* and the base of the rostral cone, reporting a partial differentiation of the dorsal rectus muscle of the eye, with thermogenic cells morphologically similar to the brown adipocytes associated with voluminous masses of periocular fat, which they defined as a “heat‐producing organ.” These authors and Carey (1982) hypothesized that these fishes possess a heating system to raise the temperature in areas of the brain and improve vision efficiency during cold water hunting. More recently, Videler *et al*. (2016) documented by magnetic resonance imaging (MRI) scans a glandular structure in a poorly mineralized, central zone near the base of the swordfish rostrum, which they defined as an “oil‐producing gland.” These authors suggested that oil was secreted on the external skin through pores to form a superhydrophobic layer lubricating the swordfish's head and increasing the swimming efficiency. To the best of this study's authors’ knowledge, no study has focused on the tissue which lies below the oil‐producing gland with fat bubbles inside, as documented in this study. Habegger *et al*. (2015) presented a similar figure, indicated simply as “cartilage” (Habegger *et al*., 2015: Figure 1, area 4) in the proximal rostrum of blue marlin (*Makaira nigricans*) and of swordfish (*X. gladius*). Nonetheless, they did not provide a detailed description or explanation of its function in rostral structure and development. Our histological images provided evidence that this tissue in the proximal cone has a close relationship with the overlying fat gland and, indeed, included a high number of fat bubbles clearly originating from the gland. This structure advanced into the rostrum, progressively reducing its sectional area until disappearing at about halfway down the full length of the rostrum. In addition, before fixation, this structure had a soft, gelatin‐like consistency; it was avascular and had no perichondrial membrane. The cellular component and the staining properties of the matrix were similar to those of the Meckel embryonic cartilage in the lower jaw of the same fish, and therefore, the authors have named it “central cartilage‐like rostral body” after its peculiar topography and histological staining properties. The volume of the fat glands occupied the majority of the space of the *X. gladius* rostral cone, as was well documented by the series of sequential macrosections carried out in this study, and by MRI in the study of Videler *et al*. (2016). Both documented the abundant production of fatty material, whereas the fat bubbles inside the cartilage rostral body suggested that (a) a secretion positive pressure is sufficient to push the fat into the tissue and (b) the compactness and fluidity of the cartilage matrix are low. Therefore, the reported histology supported the hypothesis of a forward‐directed vector force in the cartilage due to the surrounding and inextensible, calcified scaffold as a factor promoting development of the rostrum length, which is lacking in the lower jaw. The elongation of the skull with a rostral formation was also observed in some aquatic mammalians, such as delphinids and beaked whales (Cozzi *et al*., 2010), and as far as this study is concerned, the relevant observation was made that these mammals have a mesorostral cartilage, deemed homologous to the cartilaginous nasal septum of all mammals, which could have had a role in the embryonic development of rostral formation.

The rostral fat stroma of the swordfish was vascularized by a loose network of capillaries which was supported by large vessels running inside the two main parallel tunnels along the whole length of the rostrum together with myelinic nerves. Due to rostral stiffness, the bundles of myelinic fibres might not have served to innervate muscles but rather belonged exclusively to sensory nerves with only a sensory function and for which terminal receptors have not been described so far.

Despite the high degree of skeletal specialization of the swordfish rostrum, the shape of and structural differences in the lower jaw and also the differences among the other bones analysed in this teleost model, a common morphological denominator could be observed in them, *i.e*., the structuring of collagen matrix with a laminar or circular pattern. The former layout was present in the dorsal, ossifying columns of the proximal rostrum, whereas in the distal part the latter formed a densely packed texture of longitudinal, circular structures resembling those of diaphyseal bone in some mammals. A similar distribution was also observed between the outer and inner bone layers of the lower jaw and of the vertebrae. The fin rays presented a different specialization of the structural layout with a fusion‐like pattern of parallel laminae; nonetheless, circular structures were also present in the basal layer in these bones. Independently of the type of structural organization, it must be pointed out that both consisted of anosteocytic bone matrix. In particular, the circular structures were present in large number in the distal rostrum, forming together with the laminae a very compact, mixed tissue. In all the papers dealing with the rostral structure and function, this compact, mixed tissue was interpreted as being secondary osteons resulting from active remodelling (Atkins *et al*., 1986, 2014; Poplin *et al*., 1976; Shahar & Dean, 2013). Nonetheless, they better fit with the definition of “primary osteon” given in the textbook of Hall (2005) rather than with a remodelled bone layout. To the best of authors’ knowledge, in the compact rostral bone no evidence of cutting cones, of a central vascular loop and of a peripheral cement line, which are the basic features of secondary osteons, has been produced so far.

Osteogenesis and the ossification process in teleosts, in general, have been debated for a long time since Koelliker (1859) first distinguished fish species with cellular and acellular bone. The latter turned out to be the more diffuse type in the higher orders of teleosts, whose histology and mode of formation were extensively studied by Moss (1961a, 1961b). Moss also introduced the term “anosteocytic bone” and suggested that “cell withdrawal” theory explains the absence of osteocytes. Then, the coexistence of cellular and acellular bone was extensively discussed in later papers based on the phylogenesis (Lovejoy *et al*., 2004; Lynne & Parenti, 1986; Meunier, 1987, 1989). Observations of anosteocytic and cellular bone in the same fish skeleton or in a single bone were reported by Weigele and Franz‐Odendaal (2016). Hughes *et al*. (1994) in a study conducted in the teleosts Sparidae and Perciformes hypothesized that acellularity was determined by an apparent lack of osteocytic lacunae and that small osteocytes lie along the walls or in the lumen of tubules but without any clear evidence of these cells “metabolic activity.” Several questions concerning phylogenesis and osteogenesis were recently reviewed by Davesne *et al*. (2019) and the remodelling of teleosts bone by Witten and Huysseune (2009).

The ossification progression documented in this *X. gladius* study occurred in the fat stromal tissue which surrounded the already‐mineralized skeleton and filled all the lacunar spaces inside, where there was no haematopoietic tissue and no periosteal or endosteal membrane on the surface, unlike in mammalian bones. According to histological studies, the authors distinguished two phases of the process. (a) The first was characterized by scattered “foci” of fibrillogenesis forming a loose network of collagen fibre bundles between the cells that were smaller and not typically set in rows as mammalian osteoblasts; and (b) the second phase, occurring near the surface of the already‐calcified matrix, was characterized by collagen matrix compacting, calcification and embedding of the cells in the mineral scaffold. Indeed, it provided evidences that initial collagen biosynthesis and tropocollagen extrusion and fibril aggregation occurred in the fat stroma at a certain distance from the bone surface. Moreover, polarized light microscopy documented organized collagen fibre bundles far from the bone surface and their continuity through the line of calcification similar to the Sharpey fibres of the tendon insertions. This morphology can be explained only by a calcification process progressing from the already‐mineralized bone surface into a contiguous and previously organized fibrillar tissue. This pattern is different from that occurring below the periosteal, the endosteal and the cutting cone osteoblasts during secondary bone remodelling (Franz‐Odendaal *et al*., 2006; Pazzaglia *et al*., 2010, 2012).

The analysis of morphology and of the ossification process in the swordfish skeleton model can contribute to the long‐debated discussion on what has been “the enigmas of anosteocytic bone,” which is also closely related to the question of remodelling in this type of bone (Shahar & Dean, 2013). In all the examined bones from *X. gladius*, the major percentage of calcified matrix consisted of anosteocytic bone. Considering all the cells embedded in the calcified matrix as osteocytes and also the empty cell lacunae, they were scarcely represented and usually observed near the surfaces of actual or recent zones of matrix mineralization. The basic condition for cell survival inside a calcified environment such as the compact bone matrix texture is maintaining of the cell metabolic exchanges through blood and the circulating interstitial fluids. This condition is achieved in mammalian bones through the lacuno‐canalicular system connected with the intracortical and endo/periosteal vascular network (Pazzaglia *et al*., 2010, 2012; Pazzaglia & Congiu, 2013), and it is explained by the coordinated activity of the pool of osteoblasts, which in the advancement of matrix apposition are capable of establishing a connection with the processes of the osteocytes embedded in the underlying layer (Pazzaglia *et al*., 2012). Other conditions for anosteocytic bone formation (to the best of authors’ knowledge not considered so far) include the layout of the vascular network and the systolic pressure, which control both the haemodynamic conditions and the cell metabolic exchanges in the ossified tissues. The histology presented in this study documents the embedded cells and the empty cellular lacunae in the areas of active ossification and clashes with the “withdrawal theory” by Moss (1961b, 1963) and rather suggesting more that the fate of the embedded cells is necrosis or apoptosis. Recently, Ofer *et al*. (2019) hypothesized that in *D. rerio* and *Oryzias latipes* anosteocytic osteogenesis is based upon apoptosis of the entrapped cells ending up as part of the mineralized bone matrix. Nonetheless, the phenomenon of cellular lacunae or bone canal refilling is not a specific feature of fish anosteocytic bone, as it has also been documented that changes in circulation dynamics suppressed blood flow in sectors of the haversian bone system and sealed osteons were formed in mammalian bones (Congiu & Pazzaglia, 2011; Pazzaglia *et al*., 2010). Nonetheless, according to the latter hypotheses, it cannot be excluded that some osteoblasts might withdraw, leaving traces of fissures on the bone surface.

The question of bone resorption and remodeling in fish was extensively discussed in the review of Witten and Huysseune (2009) with reference to the mammalian‐like, multinucleated osteoclasts observed in Cyprinids and Salmonides (Domon *et al*., 2004; Witten *et al*., 2000) as well as with reference to the small, mononucleated osteoclasts observed in the early skeletal development of all teleosts and in later developmental stages of advanced teleosts with acellular bone. That review specified that these cells cannot be detected by using standard histological techniques and that a smooth (non‐lacunar) type of bone resorption is carried out by the small mononucleated osteoclasts, in contrast to the lacunar type of the multinuclear cells. In the present study, two mature specimens of *X. gladius* were examined using histological techniques not suited for visualizing small mononuclear osteoclasts and, therefore, no conclusions on resorption pattern in the studied swordfish could be derived from the current results. Nonetheless, in such a large‐size teleost bone, remodeling would mean substitution of a bone mass equivalent to the volume of all the rostral osteons as presently stated for the Xiphiidae and Istiophoridae families. Therefore, the mass of the removed tissue would have been considerable, and the actual remodeling should have left evidences of resorption pits detectable by SEM (Ali *et al*., 1984; Chambers, 1985), the lack of which supports the theory of unremodelled primary osteons, as put forth in this paper.

To the best of authors’ knowledge, this is the first report of a primary plexiform type of anosteocytic bone ossification in a teleost. Laminar bone was also observed in some mammals such as rabbits, pigs, wild boars, cows, calves and sheep, characterized by a mixed plexiform‐haversian texture and transition with ageing to the prevalence of the haversian model (Martiniakova *et al*., 2006; Mori *et al*., 2003). The relationship between the structural and histo‐compositional characteristics of mammalian long bones (degree of laminarity, collagen fibre orientation and mineral content) and the adaptation to the specific features of the strain have been extensively investigated in mammals (Currey, 1959; Skedros *et al*., 2003). Due to their size and shape, billfish rostra are one of the best anosteocytic bone models for correlating structural patterns and strain features (Cohen *et al*., 2012). This study therefore provides a basic set of morphological data useful for future biomechanical analyses comparing laminar and haversian bone.

### AUTHOR CONTRIBUTIONS

U.E.P., M.S. and R.M. developed the initial ideas and acquired the specimens and data; U.E.P. and Marcella Reguzzoni performed histological study and Marcella Reguzzoni and Mario Raspanti SEM study; Marcella Reguzzoni and G.Z. performed statistical analysis; U.E.P., Marcella Reguzzoni and R.M. contributed to preparation of the manuscript.

### AKNOWLEDGEMENTS

The study was carried out using a SEM microscope of the University of Insubria and the Light Microscopy facilities of the University of Brescia, thanks to a scientific research agreement between the two universities. The senior author (U.E.P.) is retired professor of Orthopaedic Surgery of the University of Brescia.

The authors thank Prof. Terenzio Congiu (University of Cagliari) for advice and his patience in discussing the SEM findings; Prof. Valeria Sibilia and Dr. Francesca Pagani (University of Milan) for help in providing swordfish samples and critical discussion on statistics; Dr. Battista Galli (Clinica Veterinaria CMV, Varese) for assistance with X‐ray; and Radiology Technician Margherita Fonsato (Spedali Civili di Brescia) for assistance with CT. Open Access Funding provided by Universita degli Studi di Brescia within the CRUI‐CARE Agreement.

## CONFLICT OF INTEREST

None of the authors have any conflict of interest.

## Supporting information


**FIGURE S7** X‐ray and CT of proximal cone of the upper jaw and rostrum and X‐rays of the lower jaw. (a) CT median longitudinal section of the proximal cone (the asterisk corresponds to the flat gland). CT, transverse sections from proximal to distal: **1** fat gland filling the whole space between the cranial vault and the floor of the upper jaw. **2–5** progressive reduction in the cone sectional area and of the fat gland volume. The floor of the upper jaw is formed by a single bone lamina extending from the left to the right edges, whereas the dorsal sector presents distinct centres of ossification; (b) X‐rays of rostrum cut segments showing the size reduction from proximal to distal and CT transverse sections documenting the ossification centres of the dorsal columns, which merged distally to form a close, cortical ring. In the two most proximal sections is still evident the central cartilage; (c) X‐rays of the lower jaw right and left branches in lateral projection. The jaw tip is taken in a‐p projection showing the branches merging to form the pointed tip

**FIGURE S8** Rostrum, transverse section (haematoxylin–eosin, bar = 100 μm). (a) Arteries and (b) myelinic nerves are present in the cellular‐adipose tissue of the two symmetrical tunnels running for the whole rostral length

**FIGURE S9** Histology of the rostrum, central cartilage (toluidine blue, bar = 100 μm): (1) spindle‐shaped chondrocytes inside cartilage. Both cell morphology and matrix staining are similar to that of the lower jaw Meckel cartilage in the same fish; (2) osmium stains the fat bubbles embedded in the cartilage matrix (osmium, bar = 1 mm); (3, 4) Alcian blue stains the matrix but not the bubbles, whereas Sirius red stains weakly the matrix and is concentrated on the borders and in the stroma of the bubbles (Alcian blue and Sirius red, bar = 1 mm)

**FIGURE S10** X‐rays of the dorsal‐fin rays in lateral projection and transverse section histology of the fourth ray basal segment (haematoxylin–eosin, bright and polarized light, bar = 50 μm): (a) the first and second rays are short and underdeveloped, and the third ray shows a single, calcified axis. From the fourth onwards the fin rays fanned out at different heights in four filaments; (b) densely packed laminae which formed in the lacunar space circular structures of anosteocytic bone (primary osteons); no evidence of bright–dark band sequence or of full “bright” osteons in polarized light. (The apical, laminar texture of the fin ray is shown in Figure 5.)

## References

[jfb15069-bib-0001] Ali, N. N. , Boyde, A. , & Jones, S. J. (1984). Mobility and resorption: Osteoclastic activity in vitro. Anatomy and Embryology (Berl), 170, 51–56. 10.1007/BF00319457 6476408

[jfb15069-bib-0002] Atkins, A. , Dean, M. N. , Habegger, M. L. , Motta, P. , Ofer, L. , Repp, F. , … Shahar, R. (2014). Remodeling in bone without osteocytes: Billfish challenge bone structure‐function paradigms. Proceeding of the National Academy of Science, 111, 16047–16052. 10.1073/pnas.1412372111 PMC4234560 25331870

[jfb15069-bib-0003] Atkins, A. , Milgram, J. , Weiner, S. , & Shahar, R. (1986). The response of anosteocytic bone to controlled loading. Journal of Experimental Biology, 218, 3559–3569. 10.1242/jeb.124073 26582932

[jfb15069-bib-0004] Betancur, R. R. , Wiley, E. O. , Arratia, G. , Acero, A. , Bailly, N. , Miya, M. , … Ortì, G. (2017). Phylogenetic classification of bony fishes. BMC Evolutionary Biology, 17(162), 1–40. 28683774 10.1186/s12862-017-0958-3 PMC5501477

[jfb15069-bib-0005] Bromage, T. G. , Goldman, H. M. , McFarlin, S. C. , Warshaw, J. , Boyde, A. , & Riggs, C. M. (2003). Circularly polarized light standards for investigations of collagen fiber orientation in bone. Anatomical Record B, 274B, 157–168. 10.1002/ar.b.10031 12964206

[jfb15069-bib-0006] Carey, F. G. (1982). A brain heater in the swordfish. Science, 216, 1327–1329. 7079766 10.1126/science.7079766

[jfb15069-bib-0007] Chambers, T. J. (1985). The pathology of the osteoclasts. Journal of Clinical Pathology, 38, 241–252. 2982920 10.1136/jcp.38.3.241 PMC499119

[jfb15069-bib-0008] Cohen, L. , Dean, M. , Shipov, A. , Atkins, A. , Monsonego‐Ornan, E. , & Shahar, R. (2012). Comparison of structural, architectural and mechanical aspects of cellular and acellular bone in two teleost fish. Journal of Experimental Biology, 215, 1983–1993. 22573778 10.1242/jeb.064790

[jfb15069-bib-0009] Congiu, T. , & Pazzaglia, U. E. (2011). The sealed osteons of cortical diaphyseal bone. Early observations revisited with scanning electron microscopy. Anatomical Record, 294, 193–198. 10.1002/ar.21309 21234993

[jfb15069-bib-0010] Cozzi, B. , Panin, M. , Butti, C. , Podestà, M. , & Zotti, M. (2010). Bone density distribution patterns in the rostrum of delphinids and beaked whales: Evidence of family‐specific evolutive traits. Anatomical Record, 293, 235–242. 10.1002/ar.21044 20027645

[jfb15069-bib-0011] Currey, J. D. (1959). Differences in the tensile strength of bone of different histological types. Journal of Anatomy, 93, 87–95. 13620620 PMC1244330

[jfb15069-bib-0012] Currey, J. D. , & Shahar, R. (2013). Cavities in the compact bone in tetrapods and fish and their effect on mechanical properties. Journal of Structural Biology, 183, 107–122. 23664869 10.1016/j.jsb.2013.04.012

[jfb15069-bib-0013] Davesne, D. , Meunier, F. J. , Schmitt, A. D. , Friedman, M. , Otero, O. , & Benson, B. J. (2019). The phylogenetic origin and evolution of acellular bone in teleost fishes: Insights into osteocyte function in bone metabolism. Biological Review, 94, 1338–1363. 10.1111/brv.12505 30924235

[jfb15069-bib-0014] De Metrio, G. , Ditrich, H. , & Palmieri, G. (1997). Heat producing organ in the swordfish (*Xiphias gladius*): A modified eye muscle. Journal of Morphology, 234, 89–96. 29852704 10.1002/(SICI)1097-4687(199710)234:1<89::AID-JMOR8>3.0.CO;2-I

[jfb15069-bib-0015] Domenici, P. , Wilson, A. D. M. , Kurvers, R. H. J. M. , Herbert‐Read, J. E. , Steffesen, J. F. , Krause, S. , … Krause, J. (2014). How sailfish use their bills to capture schooling prey. Proceedings of the Royal Society B, 281, 20140444. 24759865 10.1098/rspb.2014.0444 PMC4043100

[jfb15069-bib-0016] Domon, T. , Fukui, A. , Taniguchi, Y. , Suzuki, R. , Takahashi, S. , Yamamoto, T. , & Wakita, M. (2004). Odontoclasts in the Chinook salmon differ from mammalian odontoclasts by exhibiting a great proportion of cells with high nuclei numbers. Anatomy and Embryology, 209, 119–128. 15597190 10.1007/s00429-004-0437-7

[jfb15069-bib-0017] Ekanayake, S. , & Hall, B. K. (1987). The development of acellularity of the vertebral bone of the Japanese medaka, *Oryzias latipes* (Teleostei, Cyprinidontoidae). Journal of Morphol ogy, 193, 253–261. 10.1002/jmor.1051930304 3682003

[jfb15069-bib-0018] Fierstine, H. L. (1997). An Atlantic blue Marlin, *Makaira ingricans*, impaled by two species of billfishes (Teleostei: Istiophoridae). Bulletin of Marine Science, 61, 495–499.

[jfb15069-bib-0019] Fierstine, H. L. , & Voigt, N. L. (1996). Use of rostral characters for identifying adult billfishes (Teostei: Perciformes: Istiophoridae and Xiphiidae). Copeia, 1, 148–161.

[jfb15069-bib-0020] Franz‐Odendaal, T. A. , Hall, B. K. , & Witten, P. E. (2006). Buried alive: How osteoblasts become osteocytes. Developmental Dynamics, 235, 176–190. 16258960 10.1002/dvdy.20603

[jfb15069-bib-0021] Habegger, M. L. , Dean, M. N. , Dunlop, J. W. C. , Mullins, G. , Stokes, M. , Huber, D. R. , … Motta, P. J. (2015). Feeding in billfishes: Inferring the role of rostrum from a biomechanical standpoint. Journal of Experimental Biology, 218, 824–836. 25617457 10.1242/jeb.106146

[jfb15069-bib-0022] Habegger, M. L. , Motta, P. , Huber, D. , Pulaski, D. , Grosse, I. , & Dumont, E. (2019). Feeding biomechanics in billfishes: Investigating the role of the rostrum through finite element analysis. Anatomical Records, *303*, 44‐52. 10.1002/ar.24059. 30623594

[jfb15069-bib-0023] Hall, B. K. (2005). Bones and cartilage. Developmental and evolutionary skeletal biology. San Diego, USA: Elsevier Academic Press.

[jfb15069-bib-0024] Hughes, D. R. , Basset, J. R. , & Moffat, L. A. (1994). Histological identification of osteocytes in the allegendly acellular bone of the sea breams *Acanthopagrus australis*, *Pagrus auratus and Rhabdosargus sarba* (Sparidae, Perciformes, Teleostei). Anatomy and Embryology, 190, 163–179. 7818089 10.1007/BF00193413

[jfb15069-bib-0025] Ingleton, P. M. , Hazon, N. , Ho, P. M. W. , Martin, T. J. , & Danks, J. A. (1995). Immunodetection of parathyroid hormone‐related protein in plasma and tissues of an elasmobranch (*Scyliorhinus canicula*). General and Comparative Endocrinology, 98, 211–218. 7635275 10.1006/gcen.1995.1062

[jfb15069-bib-0026] Koelliker, A. (1859). On the different types in the microscopic structure of the skeleton of osseous fish. Proceedings of the Royal Society London, 9, 656–688.

[jfb15069-bib-0027] Lovejoy, N. R. , Iranpour, M. , & Collette, B. B. (2004). Phylogeny and jaw ontogeny of beliform fishes. Integrative Comparative Biology, 44, 366–377. 21676722 10.1093/icb/44.5.366

[jfb15069-bib-0028] Lynne, R. , & Parenti, F. L. S. (1986). The phylogenetic significance of bone types in euteleost fishes. Zoological Journal of the Linnean Society, 87, 37–51.

[jfb15069-bib-0029] Martiniakova, M. , Grosskopf, B. , Omerlka, R. , Vandrakova, M. , & Bauerova, M. (2006). Histological study of compact bone tissue in some mammals: A method for species determination. Internation Journal of Osteoarcheology, 17, 82‐90. 10.1002/oa.856.

[jfb15069-bib-0030] Mc Gowan, C. (1988). Differential development of the rostrum and mandible of the swordfish (*Xiphias gladius*) during ontogeny and its possible functional significance. Canadian Journal of Zoology, 66, 486–503.

[jfb15069-bib-0031] Meunier, F. J. (1987). Os cellulaire, os acellulare et tissus dérivés chez les Osteichthyens: les phénoméns de l' acellularisation e t de la perte de minéralisation. Annals of Biology, 26, 201–233.

[jfb15069-bib-0032] Meunier, F. J. (1989). The acellularization process in osteoichthyan bone. In H. Splechtna & H. Hilgers (Eds.), Trends in vertebrate morphology (pp. 443–446). Stuttgart: Gustav Fisher Verlag.

[jfb15069-bib-0033] Mori, R. , Kodaka, T. , Sano, T. , Yamagishi, N. , Asari, M. , & Naito, Y. (2003). Comparative histology of the laminar bone between young calves and foals. Cells Tissue Organs, 175, 43–50. 10.1159/000073436 14605494

[jfb15069-bib-0034] Moss, M. L. (1961a). Osteogenesis of acellular teleost fish bone. American Journal of Anatomy, 108, 99–104.

[jfb15069-bib-0035] Moss, M. L. (1961b). Studies of the acellular bone of teleost fish I. Morphological and systematic variations. Acta Anatomica, 46, 343–462. 14476542

[jfb15069-bib-0036] Moss, M. L. (1963). The biology of acellular teleost bone. Annals of the New York Academy of Science, 109, 337–350. 10.1111/j.1749-6632.1963.tb13475.x 13936210

[jfb15069-bib-0037] Near, T. J. , Eytan, R. I. , Dornburg, A. , Kuhn, K. L. , Moore, J. A. , Davies, M. P. , … Smith, W. L. (2012). Resolution of ray‐finned fish phylogeny and timing of diversification. Proceedings of the National Academy of Sciences, 109, 13698–13703. 10.1073/pnas.1206625109 PMC3427055 22869754

[jfb15069-bib-0038] Ofer, L. , Dumont, M. , Rack, A. , Zaslansky, P. , & Sahar, R. (2019). New insights into the processo f osteogenesis of anosteocytic bone. Bone, 125, 61–73. 31085351 10.1016/j.bone.2019.05.013

[jfb15069-bib-0039] Pazzaglia, U. E. , & Congiu, T. (2013). The cast imaging of the osteon lacunar‐canalicular system and the implications with functional models of intracanalicular flow. Journal of Anatomy, 222, 193–202. 23082756 10.1111/joa.12004 PMC3632224

[jfb15069-bib-0040] Pazzaglia, U. E. , Congiu, T. , Marchese, M. , Zarattini, G. , & Dell'Obo, C. (2012). The canalicular system and the osteoblast domain in human secondary osteons. Anatomy Histology Embryology, 41, 410–418. 10.1111/j.1439-0264.2012.01150.x 22469429

[jfb15069-bib-0041] Pazzaglia, U. E. , Zarattini, G. , Giacomini, D. , Rodella, L. , Menti, A. M. , & Feltrin, G. (2010). Morphometric analysis of the canal system of cortical bone. An experimental study in the rabbit femur carried out with standard histology and micro‐CT. Anatomia Histologia Embryologia, 39, 17–26. 19874276 10.1111/j.1439-0264.2009.00973.x

[jfb15069-bib-0042] Poplin, C. , Poplin, F. , & Ricqles, A. D. (1976). Some anatomical and histological particularities of rostrum of swordfish (*Xiphias gladius)* . Comptes Rendus Hebdomadaires des Sciences de l' Academie des Sciences, Serie D, 282(11), 1105–1108.

[jfb15069-bib-0043] Schmidt, F. N. , Zimmermann, E. A. , Walsh, F. , Plumeyer, C. , Schaible, E. , Fiedler, I. A. K. , … Busse, B. (2019). On the origins of fracture toughness in advanced teleosts: How the swordfish sword's bone structure and composition allow for slashing under water to kill or stun prey. Advanced Science (Weinh), 6, 1900287. 10.1002/advs.201900287. PMC6662059 31380168

[jfb15069-bib-0044] Shahar, R. , & Dean, M. N. (2013). The enigmas of bone without osteocytes. 2 BoneKEy Reports, 2, 343. 10.1038/bonekey.2013.77. 24422081 PMC3692265

[jfb15069-bib-0045] Simmons, D. J. (1971). Calcium and skeletal tissue physiology in teleost fishes. Clinical Orthopaedics and Related Research, 76, 244–280. 4931061 10.1097/00003086-197105000-00031

[jfb15069-bib-0046] Skedros, J. G. , Dayton, M. R. , Sybrowsky, C. L. , Bloebaum, R. D. , & Bachus, K. N. (2003). Are uniform regional safety factors an objective of adaptive modeling/remodeling in cortical bone? Journal of Experimental Biology, 206, 2431–2439. 12796459 10.1242/jeb.00466

[jfb15069-bib-0047] Videler, J. J. , Heydar, D. , Snoek, R. , Hoving, H.‐J. T. , & Szabo, B. G. (2016). Lubrificating the sword fish head. Journal of Experimental Biology, 219, 1953–1956. 27385753 10.1242/jeb.139634

[jfb15069-bib-0048] Weigele, J. , & Franz‐Odendaal, T. A. (2016). Functional bone histology of zebra fish reveals two types of endochondral ossification, different types of osteoblasts clusters and a new bone type. Journal of Anatomy, 229, 92–103. 27278890 10.1111/joa.12480 PMC5341596

[jfb15069-bib-0049] Weiss, R. E. , & Watabe, N. (1979). Studies on the biology of fish bone. III. Ultrastructure of osteogenesis and resorption in osteocytic (cellular) and anosteocytic (acellular) bones. Calcified Tissue International, 28, 43–56. 115551 10.1007/BF02441217

[jfb15069-bib-0050] Witten, P. E. , Hansen, A. , & Hall, B. K. (2001). Features of mono‐ and multinucleated resorbing cells of the zebrafish *Danio rerio* and their contribution to skeletal development, remodeling and growth. Journal of Morphology, 250, 197–207. 11746460 10.1002/jmor.1065

[jfb15069-bib-0051] Witten, P. E. , & Huysseune, A. (2009). A comparative view on mechanisms and function of skeletal remodeling in teleost fish with acellular bone, with special emphasis on osteoclasts and their function. Biological Review of the Cambridge Philosophical Society, 84, 315–346. 10.1111/j.1469-185X.2009.00077.x 19382934

[jfb15069-bib-0052] Witten, P. E. , Villwock, W. , Peters, N. , & Hall, B. K. (2000). Bone resorption and bone remodeling in juvenile carp (*Cyprinus carpio*). Journal of Applied Ichthyology, 16, 254–261.

