# Supplementary figures and images for: The complex rostral morphology and the endoskeleton ossification process of two adult samples of Xiphias gladius (Xiphiidae)
Source: J Fish Biol. 2022 May 16;101(1):42–54. doi: 10.1111/jfb.15069 (PMC9545449; doi:10.1111/jfb.15069)

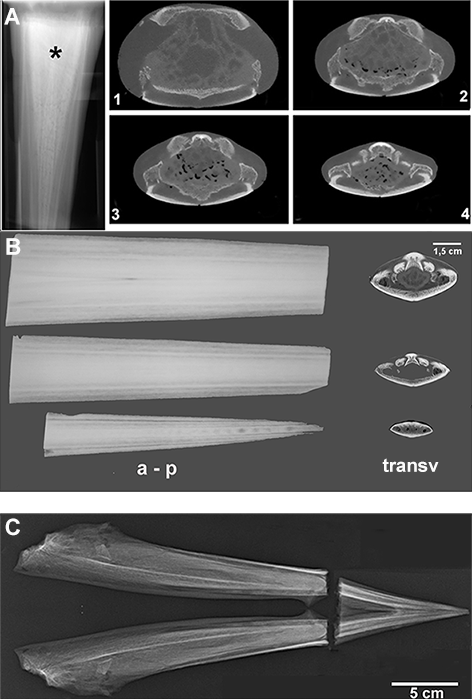

Supplement: Supplementary file 1 — FIGURE S7 X‐ray and CT of proximal cone of the upper jaw and rostrum and X‐rays of the lower jaw. (a) CT median longitudinal section of the proximal cone (the asterisk corresponds to the flat gland). CT, transverse sections from proximal to distal: 1 fat gland filling the whole space between the cranial vault and the floor of the upper jaw. 2–5 progressive reduction in the cone sectional area and of the fat gland volume. The floor of the upper jaw is formed by a single bone lamina extending from the left to the right edges, whereas the dorsal sector presents distinct centres of ossification; (b) X‐rays of rostrum cut segments showing the size reduction from proximal to distal and CT transverse sections documenting the ossification centres of the dorsal columns, which merged distally to form a close, cortical ring. In the two most proximal sections is still evident the central cartilage; (c) X‐rays of the lower jaw right and left branches in lateral projection. The jaw tip is taken in a‐p projection showing the branches merging to form the pointed tip [file JFB-101-42-s003.tif]

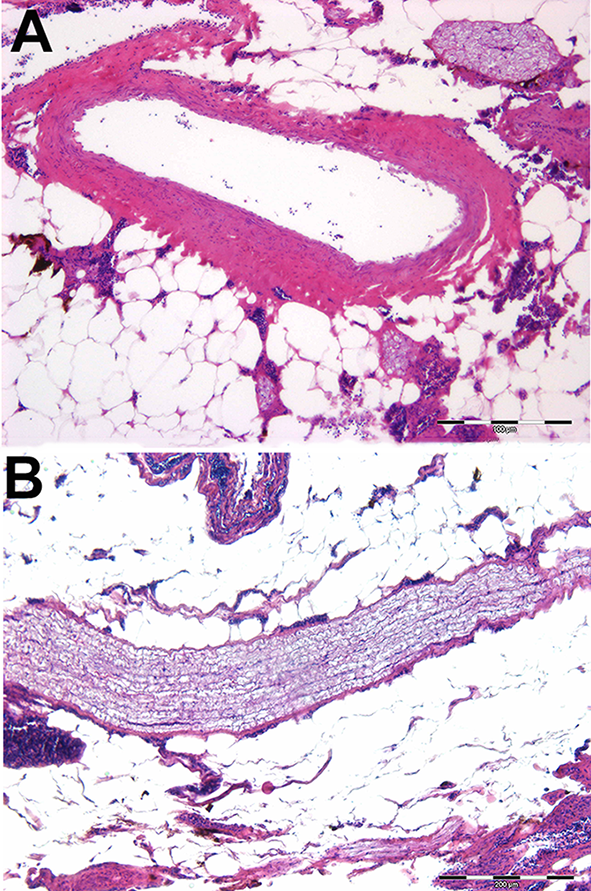

Supplement: Supplementary file 2 — FIGURE S8 Rostrum, transverse section (haematoxylin–eosin, bar = 100 μm). (a) Arteries and (b) myelinic nerves are present in the cellular‐adipose tissue of the two symmetrical tunnels running for the whole rostral length [file JFB-101-42-s001.tif]

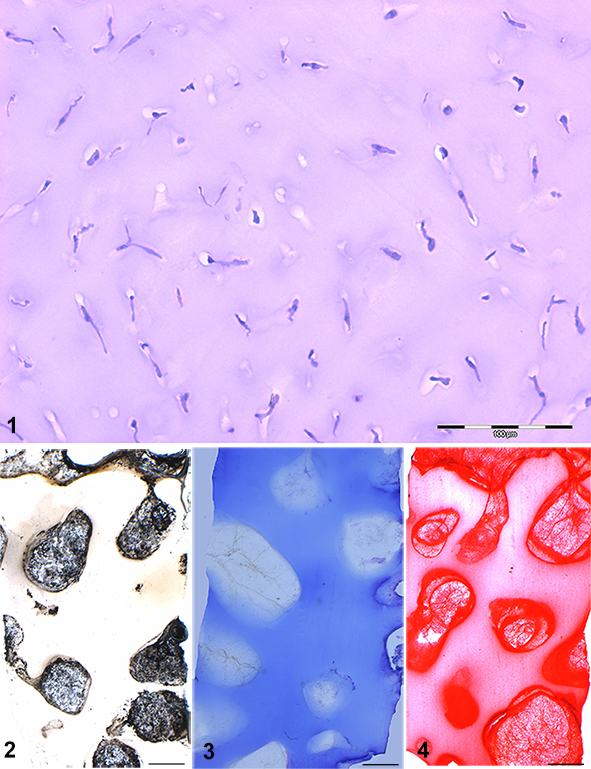

Supplement: Supplementary file 3 — FIGURE S9 Histology of the rostrum, central cartilage (toluidine blue, bar = 100 μm): (1) spindle‐shaped chondrocytes inside cartilage. Both cell morphology and matrix staining are similar to that of the lower jaw Meckel cartilage in the same fish; (2) osmium stains the fat bubbles embedded in the cartilage matrix (osmium, bar = 1 mm); (3, 4) Alcian blue stains the matrix but not the bubbles, whereas Sirius red stains weakly the matrix and is concentrated on the borders and in the stroma of the bubbles (Alcian blue and Sirius red, bar = 1 mm) [file JFB-101-42-s002.tif]

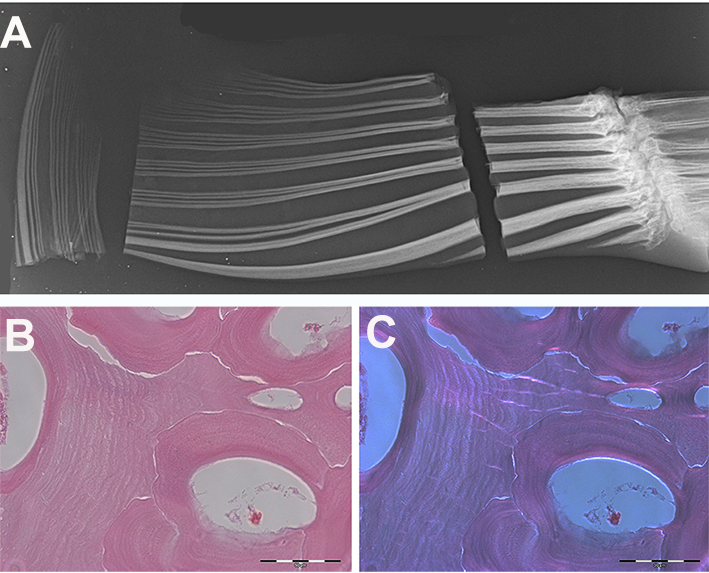

Supplement: Supplementary file 4 — FIGURE S10 X‐rays of the dorsal‐fin rays in lateral projection and transverse section histology of the fourth ray basal segment (haematoxylin–eosin, bright and polarized light, bar = 50 μm): (a) the first and second rays are short and underdeveloped, and the third ray shows a single, calcified axis. From the fourth onwards the fin rays fanned out at different heights in four filaments; (b) densely packed laminae which formed in the lacunar space circular structures of anosteocytic bone (primary osteons); no evidence of bright–dark band sequence or of full “bright” osteons in polarized light. (The apical, laminar texture of the fin ray is shown in Figure 5.) [file JFB-101-42-s004.tif]
